# Developing and Implementing an Occupation-Based Practice Service Delivery Model for Stroke Survivors in a Rural Thai Community Rehabilitation Center: Mixed Methods Feasibility Study

**DOI:** 10.2196/91118

**Published:** 2026-09-21

**Authors:** Sorasak Suwan, Anuchart Kaunnil, Peeraya Munkhetvit, Hataichanok Apikomonkon, Sarinya Sriphetcharawut

**Affiliations:** 1 Occupational Therapy Department, Faculty of Associated Medical Sciences, Chiang Mai University, 110 Intawaroros Rd., Sripoom Subdistrict, Mueang District, Chiang Mai, 50200, Thailand, 66 846160504, 66 53936042

**Keywords:** occupation-based practice, stroke, community-based rehabilitation, service delivery, occupational therapy

## Abstract

**Background:**

Occupation-based practice (OBP) has been demonstrated to enhance occupational performance and goal attainment in stroke rehabilitation. However, existing evidence is predominantly derived from hospital-based settings, with limited investigations into OBP implementation in community contexts. In Thailand, particularly in rural areas, there remains a need to establish effective OBP service delivery models and to evaluate their outcomes across diverse client populations receiving community-based rehabilitation services.

**Objective:**

This study aimed to develop an OBP service delivery model for community-based stroke rehabilitation in the Doi Lor community, Chiang Mai Province, Thailand, and to evaluate its effects on occupational performance, goal attainment, and quality of life among community-dwelling stroke survivors. Additionally, the study explored the occupational therapist’s experience and perceptions of implementing the model, particularly the practicality of implementation in long-term community-based occupational therapy services.

**Methods:**

This study used an embedded mixed methods feasibility design comprising a 1-group pretest-posttest intervention study and a qualitative descriptive interview. An OBP service delivery model for community-based stroke rehabilitation was developed based on the Dynamic Model of Occupation-Based Practice; the Occupational Therapy Practice Framework, Fourth Edition; and evidence-based stroke rehabilitation principles. The model was implemented by a trained occupational therapist with 7 stroke survivors at a rural community rehabilitation center over 14 sessions spanning 7 weeks. Quantitative outcomes were assessed before and after the intervention using the Canadian Occupational Performance Measure, goal attainment scaling, and the World Health Organization Quality of Life–Brief Version. Qualitative data were collected through a semistructured interview with the occupational therapist to explore experiences of implementing the OBP service delivery model.

**Results:**

In the quantitative analysis, participants demonstrated statistically significant improvements in occupational performance and satisfaction as measured by the Canadian Occupational Performance Measure (*P*=.02), higher levels of goal attainment on the goal attainment scaling (*P*=.02), and improved overall quality of life as assessed by the World Health Organization Quality of Life–Brief Version (*P*=.02). Qualitative findings from the occupational therapist interview indicated 2 themes, including facilitating client-centered outcomes through OBP implementation and enhancing professional confidence and supporting the practical implementation of OBP in community-based practice. The occupational therapist described the model as practical and feasible and reported increased confidence in delivering occupation-based interventions. The findings also highlighted the potential applicability of the model within long-term, community-based occupational therapy services.

**Conclusions:**

This preliminary study suggests that an OBP service delivery model grounded in therapist preparation, client-centered goal setting, and outcome-focused intervention may support improvements in occupational performance, goal attainment, and quality of life among stroke survivors receiving community-based rehabilitation. This approach may contribute to strengthening long-term stroke care, particularly in rural communities where access to hospital-based rehabilitation services is limited.

## Introduction

Stroke remains the world’s second most common cause of death and the third most common cause of both death and disability [1]. In Thailand, data on stroke incidence indicate a rising trend in incidence rates and chronic stroke cases [2]. Many stroke survivors are unable to perform activities of daily living (ADL) due to mental, emotional, cognitive, and physical disabilities [3]. Consequently, a significant proportion of stroke survivors do not adequately regain their functional abilities, daily life skills, or quality of life after stroke [4]. On average, their quality of life was significantly reduced 1 year after their stroke, despite relatively mild levels of impairment [5].

Occupational therapists play a key role in stroke rehabilitation by focusing on occupational performance in everyday life and social participation. Their holistic approach considers multiple factors influencing occupational performance and participation [6]. Occupational therapy service delivery emphasizes occupation as the fundamental element of therapy, allowing clients to engage in meaningful occupations that enhance their performance, health, and well-being [7]. Additionally, occupation-based practice (OBP) is an approach used by occupational therapists to improve clients’ occupational performance, which involves assessment, intervention, and outcome evaluation [8]. Strong evidence indicates that OBP benefits stroke clients in areas such as mobility, occupational performance, mental health, quality of life, and overall well-being, although most studies have been conducted in hospital settings [9-16].

Despite these positive outcomes, hospital-based OBP faces challenges, including constraints of time, space, and equipment [17], as well as staff shortages and high caseloads [18]. Additionally, OBP’s holistic, client-centered approach may conflict with hospital policies, service models, and expectations that prioritize medical conditions and management [19]. Many occupational therapists remain unclear about OBP principles and implementation [19,20], even though its use reflects the profession’s identity of using purposeful and meaningful activities with clients [8].

In Thailand, the application of occupation-based approaches has only recently begun to emerge as a focus of research examining the effectiveness of occupational therapy interventions. For example, Pongtham et al [11] investigated the effects of task-oriented occupational therapy intervention on upper-extremity function among individuals with stroke. In addition, a mixed methods study conducted in 2021 reported that Thai occupational therapists strongly support the use of OBP. The findings further indicated that clinical practice and occupational therapy education should be grounded in this approach and embedded in university curricula for future occupational therapists [19].

The Faculty of Associated Medical Sciences at Chiang Mai University places a strong emphasis on community academic service as part of its commitment to giving back to society. In line with this mission, the Occupational Therapy Department aims to support sustainable development within target communities. Therefore, this study focused on the Doi Lor Subdistrict in Doi Lor District, Chiang Mai Province—an area well-suited for such collaborative-based rehabilitation. Environmental factors play a significant role in implementing OBP, especially when therapy occurs in the client’s home and/or community rather than in a hospital or other unfamiliar settings, which may limit occupational engagement and participation [21]. Community-based occupational therapy for stroke survivors requires coordinated efforts across multiple sectors. Strengthening the competencies of community-based occupational therapists in occupation-based assessment and intervention represents an important investment in ensuring effective and sustainable rehabilitation practices. The outcomes tend to be more responsive to clients’ needs, particularly in enabling them to perform meaningful daily occupations independently. This approach also enhances participation with families and the broader community. Significantly, it holds strong potential for further development as a cost-effective approach to occupational therapy for stroke rehabilitation.

Chiang Mai, the capital city of Northern Thailand, is characterized by a complex interplay of environmental, cultural, and socioeconomic factors that influence health and rehabilitation service delivery. The Doi Lor Subdistrict, situated in a rural area of Chiang Mai Province, is made up of 26 villages with a total population of 12,227 residents (5982 males and 6245 females). Approximately 70% of the population is engaged in agricultural occupations, with primary crops including longan, mango, rice, cantaloupe, tomato, and pumpkin; longan represents the area’s main export commodity [22]. By 2024, a total of 95 individuals with stroke had been officially registered in the Doi Lor community, including 5 individuals aged 40 to 49 years, 17 individuals aged 50 to 59 years, and 73 individuals aged 60 years and older [23]. The subdistrict includes 1 district hospital, 3 subdistrict health-promoting hospitals, and 1 community rehabilitation center, staffed by a single occupational therapist. The substantial number of patients and the need for their reintegration into society highlight the importance of the physical, mental, and economic aspects of each family. Consequently, occupational therapists have to apply interventions that are aligned with the specific contexts of the participants to enhance their ability to engage independently in occupations.

The use of OBP in Thai occupational therapy still requires further research to guide service models for clients with stroke and to expand the evidence for outcomes associated with this approach. Such work is essential for illustrating the transformative impact that meaningful daily occupations can have on health, participation, and quality of life. This aligns with the core philosophy of the occupational therapy profession, which holds that well-being is deeply connected to opportunities for engaging in occupations that are meaningful, valuable, desired, or expected in one’s life [24]. Accordingly, the purpose of this preliminary mixed methods study was to develop an OBP service delivery model for community-based stroke rehabilitation. Specifically, the study aimed to (1) evaluate the effects of the OBP service delivery model on occupational performance, goal attainment, and quality of life among community-dwelling stroke survivors, and (2) explore the occupational therapist’s experiences and perceptions of implementing the model, particularly its feasibility, practicality, and potential for further application in long-term community-based occupational therapy services.

## Methods

### Research Design

This study used an embedded mixed methods feasibility design [25] to develop, implement, and conduct a preliminary evaluation of an OBP service delivery model for community-based stroke rehabilitation. The quantitative strand consisted of a preexperimental 1-group, pretest-posttest intervention study to evaluate preliminary client outcomes, while the embedded qualitative strand involved a descriptive interview with the occupational therapist to explore implementation experiences and assess the feasibility of the OBP service delivery model within a rural community rehabilitation setting.

The study was conducted in 3 sequential phases. Phase 1 involved developing a contextually relevant OBP service delivery model for rural community-based stroke rehabilitation and training a community-based occupational therapist to achieve competency in implementing the model. Phase 2 involved implementing the OBP service delivery model with community-dwelling stroke survivors by the trained occupational therapist, followed by a preliminary evaluation of its effects on occupational performance, goal attainment, and quality of life. Phase 3 consisted of an in-depth qualitative interview with the same occupational therapist who implemented the intervention to explore her experiences and perceptions of implementation, particularly regarding the feasibility, practicality, and potential for further application of the OBP service delivery model within the long-term rural community rehabilitation context.

### Ethical Considerations

Ethical approval was granted by the Human Research Ethics Unit Committee of the Faculty of Associated Medical Sciences, Chiang Mai University, Chiang Mai, Thailand (reference number AMSEC-67EX-031). Participants provided written informed consent before enrolling in this study. All identifiable data were kept confidential, anonymized prior to data analysis, and securely stored on access-restricted devices. Quantitative participants received THB 100 (THB 1=US $0.03 as of October 14, 2025) each time they accessed services as compensation for their time, which included pretreatment and posttreatment assessments. This compensation was provided for a total of 16 sessions, amounting to THB 1600 (THB 1=US $0.03 as of October 14, 2025). The occupational therapist participant received THB 20,000 (THB 1=US $0.03 as of December 2, 2025) as compensation for the delivery of OBP services to 7 stroke clients in phase 2 and for being interviewed in phase 3.

### Participants

The participants were invited to participate in the study using purposive sampling based on the inclusion criteria in this study, and they received occupational therapy services at the Doi Lor Community Rehabilitation Center from October 2024 to October 2025. Inclusion criteria were (1) age between 18 and 75 years; (2) a clinical diagnosis of stroke at least 6 months prior to enrollment; and (3) willingness to participate in the study. Exclusion criteria included the presence of perceptual or cognitive impairments, depressive symptoms, a high risk of falls, or other medical or neurological complications that could interfere with participation in the intervention or outcome assessments. As this study was designed as a preliminary investigation, a formal sample size or power calculation was not conducted.

Inclusion criteria for participants (occupational therapists) who voluntarily participated in phases 2 and 3 were as follows: (1) possession of a valid occupational therapy license, (2) a minimum of 1 year of experience delivering occupational therapy services to patients with physical dysfunction, and (3) willingness to participate in this study.

### Outcome Measures

The Canadian Occupational Performance Measure (COPM), the goal attainment scaling (GAS), and the World Health Organization Quality of Life–Brief Version (WHOQOL-BREF) were used to measure the outcomes. The COPM is a client-centered and client-perceived measure that emphasizes the client’s ability and satisfaction with performance of daily activities. The participant is asked to identify occupational performance problems, which are divided into 3 areas: self-care, productivity, and leisure. For each problem, the client rates its importance on a scale of 1 to 10. After this step, participants choose up to 5 problems that are most important to them. They then rate their current performance and satisfaction for each selected problem using a scale where 10 indicates extreme ability or satisfaction and 1 indicates not able to do it at all or not satisfied at all [26]. The average scores of performance and satisfaction were used to analyze the comparison of pretests and posttests for each participant. This measure was examined for test-retest reliability in Thai stroke clients, demonstrating strong performance in daily activities (*r*=0.879; *P*<.001) and satisfaction (*r*=0.956; *P*<.001) [27].

GAS is an assessment instrument used to measure the therapeutic outcomes of individual service users and enables each service user to evaluate their own outcomes. However, the scoring is standardized to enable statistical analysis. The primary characteristic of using GAS is the prioritization of criteria for successful outcomes for each individual. Before beginning therapy, the therapist, who rates the score on each goal, will consult the client and their family to guarantee that all parties have realistic expectations, thereby ensuring that the established objectives are feasible. For each goal, the score is divided into a 5-point scale. If participants achieve their goal, the score is 0. If participants achieve their goal better than the expected level, they receive a score of +1 (somewhat better) or +2 (much better). If they achieve goals worse than the expected level, the score level is −1 (somewhat worse) or −2 (much worse). The overall GAS score for each participant was calculated as a T-score [28] for comparison between pretests and posttests.

The Thai version of the World Health Organization Quality of Life–Brief Version (WHOQOL-BREF-THAI) is a self-assessment tool for quality of life that measures an individual’s perception of life within the context of culture, society, and the environment, in relation to their objectives, expectations, standards, and interpersonal interactions. It has been modified from the World Health Organization Quality of Life (WHOQOL)–100. The WHOQOL-BREF-THAI consists of 26 items, including 2 categories of inquiries: perceived objective inquiries and self-reported subjective inquiries. The 4 components that contribute to quality of life include the physical, psychological, social, and environmental aspects. These 26 items included 23 positively worded items and 3 negatively worded items. Negatively worded items are reverse scored prior to calculating the total score. The participant must choose the most appropriate option and respond to each question on a scale of 1 to 5, in which the definitions of the scores will differ between positive and negative questions. For score interpretation, overall scores were divided into 3 levels: poor, moderate, and excellent quality of life [29]. In this study, we analyzed the comparison of pretest and posttest scores for each participant using their overall average score.

### Procedure

An occupational therapist who volunteered to participate in this study was informed of the study’s purpose and methodology.

#### Phase 1: Development of the OBP Service Delivery Model and Preparation

This involved preparing the therapist and designing a service delivery model that aligns with the occupational therapy service context at the Doi Lor Subdistrict Administrative Organization Rehabilitation Center. The research team conducted a needs assessment through an interview with an occupational therapist. The interview focused on therapeutic intent, the purposeful value of participation, routine service delivery practices to identify gaps, and the daily occupational needs of clients in the Doi Lor Subdistrict area. Information obtained from the interview informed the development of the expected learning objectives and training framework and guided the design of the training content and timetable. Moreover, the following four steps were implemented: (1) pretest; (2) educational training with workshops focusing on OBP and task-oriented approaches; (3) posttest; and (4) designing the services along with developing a service delivery framework. Details of each step were as follows: the occupational therapist was pretested on her understanding of knowledge related to OBP, the occupational therapy process, and stroke management, focusing on a task-oriented approach. Once the intervention was completed, the posttest was administered again. The occupational therapist’s understanding of knowledge regarding OBP was assessed in the pretests and posttests via a Google Form containing 20 true-false questions. The 16-hour online training program was developed by the research team for the occupational therapist, who has a dependable internet connection through government-supported facilities, including community rehabilitation centers and associated clinical environments. This online educational training package covers core components, including the concepts of OBP, occupational profiles for stroke clients, the COPM, GAS, occupation-based goals, designing and analyzing occupations for stroke clients, task-oriented approaches, and documentation. During the online training, the first author facilitated a 1-hour workshop focusing on how to use the occupational profile and how to apply a task-oriented approach in practice. This ensured that the occupational therapist understood how to use this knowledge in developing the intervention for participants.

The research team developed a structured OBP service delivery model tailored to the rural context. The occupational therapist provided contextual information on existing service delivery practices, characteristics of the local setting, and participant needs. This information, together with other relevant information, was used by the research team to ensure that the model was appropriate for the community. In this study, the service delivery model described the organization, implementation, provision, and evaluation of occupational therapy services focusing on OBP to effectively address stroke clients’ needs. It was explicitly aligned with the Occupational Therapy Practice Framework: Domain and Process, Fourth Edition (OTPF-4), particularly the occupational therapy process [7], and was grounded in the OBP constructs proposed by Psillas and Stav [21]. Moreover, it represented a dynamic, nonlinear, and iterative process, emphasizing authentic occupation, engaged participation that is meaningful and purposeful, and applies therapeutic intent across all occupational processes, including evaluation, intervention, and outcomes.

#### Phase 2: Preliminary Evaluation of the Implementation of an OBP Delivery Model for Community-Dwelling Stroke Survivors

The service delivery emphasized home- and community-based occupational therapy services by integrating the needs of clients, caregivers, or family members and accommodating OBP practitioners. It aimed to provide a standardized and flexible OBP-guided service model while also supporting competency-based training for the occupational therapist, who was the sole practitioner working in this area. This service model was designed to monitor outcomes and establish service guidelines that could be applied in routine practice. During the process, the research team served as coaches by assisting with activity planning, administering and modifying interventions, and providing guidance or consultation to ensure that service delivery aligned with the study’s purpose and the specific occupational issues and goals of each participant. It also included discussing relevant information, offering suggestions, and monitoring the delivery of services.

During the evaluation and outcomes process, in accordance with the OTPF-4, an occupational therapist who was not involved in delivering the OBP service delivery model administered the COPM, GAS, and WHOQOL-BREF to participants in person, using paper-based assessment forms, before the intervention phase. The COPM and GAS were done to establish the 3 most important individual goals, and the intervention was subsequently tailored to address each client’s specific goals and needs. The targeted occupational goals included all 3 areas of the COPM. For example, a stroke participant chose ADL as the most important activity, which is classified as the self-care area by the COPM, while the second activity was writing his name, which is categorized as the productivity area. Furthermore, the intervention was developed by integrating the concept of the occupation-based community rehabilitation program [10] with a task-oriented approach. Within the task-oriented approach, movement is performed in various ways, functional goals are established, and client-centered practices are emphasized. Intervention characteristics include frequent repetition, manipulation of real-life objects, and practice within context-specific environments that allow for progression and variability. Feedback on performance is incorporated, and activities engage multiple movement planes. The approach also includes individualized training loads, total skill practice, randomized and distributed practice, and the use of bimanual tasks [30]. Following the occupation-based goal setting, each client received the intervention twice weekly for 7 weeks, with each session lasting 60 minutes, for a total of 14 sessions. Participants’ homes and the community rehabilitation center were used to deliver the intervention.

As part of OBP treatment, some methods may involve completing or simulating an aspect of occupation [21]. For example, practicing grasping objects can prepare participants for cooking activities, while practicing walking around and transitioning from sitting to standing can support community mobility. During this process, the occupational therapist may use clinical judgment to provide interventions to support occupational engagement, such as strengthening exercises, range of motion exercises, and spasticity management, particularly when participants experienced condition-related symptoms while participating in targeted occupations. One week after the completion of each client’s final intervention session, outcomes were reassessed using the COPM, GAS, and WHOQOL-BREF. In addition, the occupational therapist was interviewed to reflect on her learning experience from the educational training and workshops focused on OBP, as well as her experience implementing OBP with all 7 clients.

#### Phase 3: Interviews with an Occupational Therapist After the Use of the OBP Service Delivery Model

To better understand the therapist’s experiences of implementing the OBP service delivery model, the last author (S Sriphetcharawut) conducted a semistructured interview with the occupational therapist who implemented the intervention. The interview questions (as provided in Multimedia Appendix 1) explored the therapist’s experiences, perceptions, and contextual factors influencing the use of OBP in stroke rehabilitation. The interview was conducted via telephone at a mutually agreed-upon time outside working hours, was audio-recorded, and was transcribed verbatim.

### Data Analysis

Quantitative data analysis was conducted using SPSS (version 26.0; IBM Corp). The general variables for the participant group, including age, sex, education level, disease diagnosis, and duration of disease, were analyzed using descriptive statistics. The Wilcoxon signed rank test was used to analyze the significance of the average scores for COMP, GAS, and WHOQOL-BREF before and after the intervention. Sign tests were conducted to examine the consistency of change direction across participants. Furthermore, qualitative data were analyzed using content analysis [31], based on the occupational therapist’s reflections, with representative quotations presented to illustrate the findings.

The interview transcript was analyzed using conventional qualitative content analysis (Table S3 in Multimedia Appendix 2), and the analysis was conducted manually, as the qualitative dataset comprised a single participant interview. The audio-recorded interview was transcribed verbatim, and the transcript was read repeatedly by the last author (S Sriphetcharawut) and second author (AK) to achieve data immersion and a comprehensive understanding of the participant’s experiences. Meaning units relevant to the occupational therapist’s experiences of implementing the OBP service delivery model for community-dwelling stroke survivors were identified and condensed while preserving their core meaning and were assigned descriptive codes. The codes were independently compared for similarities and differences, grouped, and then inductively organized into subcategories and broader categories. The categories were continuously reviewed, compared, and refined to capture the latent meaning underlying the participant’s experiences while maintaining a close connection to the original data.

S Sriphetcharawut and AK independently conducted the coding and categorization and subsequently compared their coding and interpretations. Any discrepancies were discussed with the research team through peer debriefing until consensus was reached. The final categories and interpretations were reviewed by S Sriphetcharawut, AK, and S Suwan to ensure that they were grounded in the interview data and accurately reflected the participant’s experiences. To enhance the trustworthiness of the findings, the transcript was checked against the audio recording to verify transcription accuracy, and representative quotations were retained to illustrate each category and demonstrate the connection between the original data and the analytical interpretations. After developing identifying codes and themes, the translation process began. Selected portions of the verbatim transcript containing identifying codes and themes were translated from Thai into English by the last author and checked for language equivalency by the second author. The final English translation was proofread by a native English editor.

## Results

### Development of OBP the Service Delivery Model

**Figure 1.**
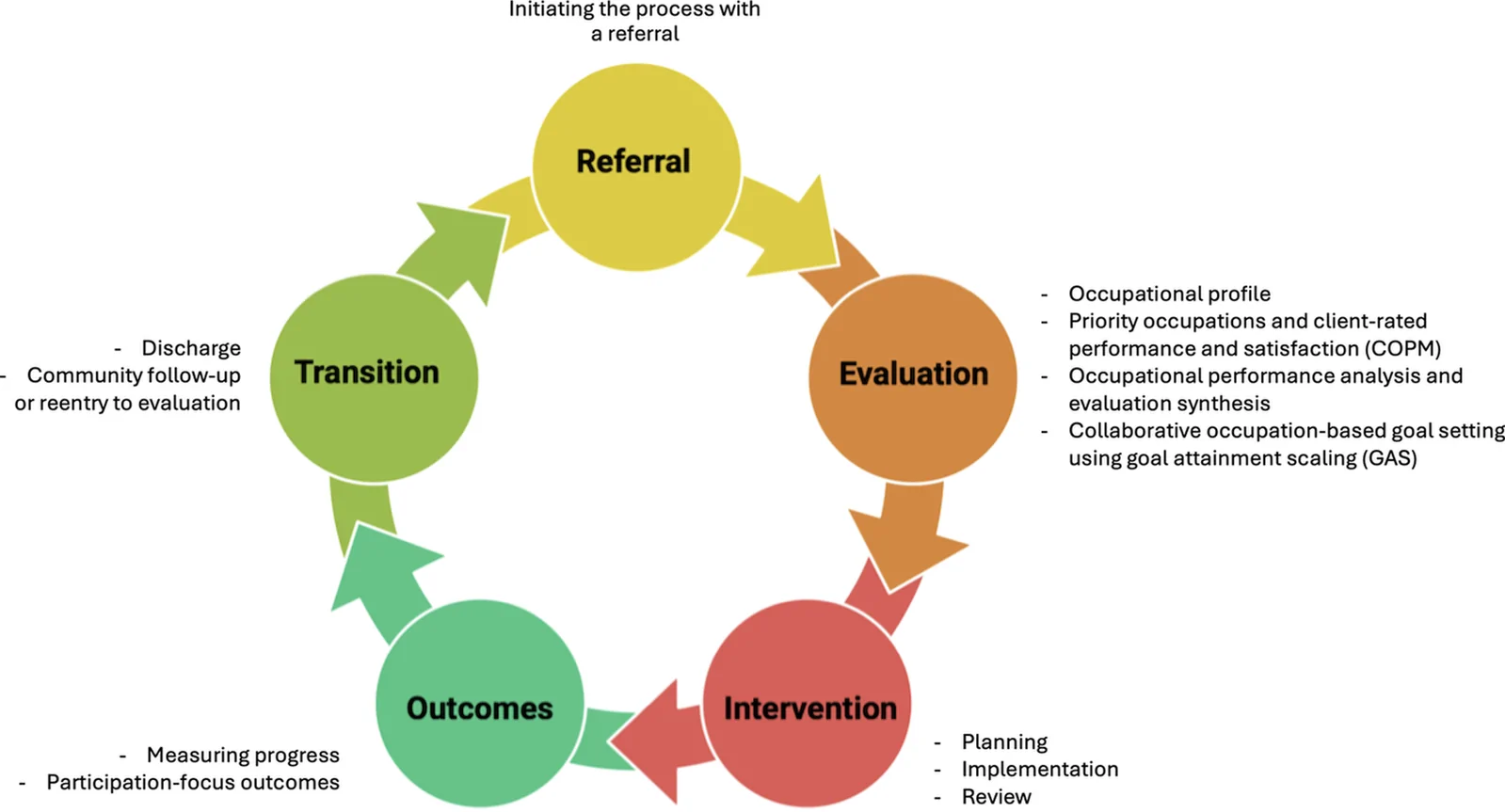
The occupation-based practice service delivery model for stroke clients at the Doi Lor Subdistrict Administrative Organization Rehabilitation Center.

The purpose of this preliminary study was to develop an OBP service delivery model aimed at enhancing occupational performance, promoting personal goals, and improving the quality of life for stroke clients in long-term, community-based rehabilitation settings. The procedure for the OBP service delivery model is illustrated in Figure 1.

The OBP service delivery model for individuals with stroke in the Doi Lor Subdistrict community (Figure 1) is implemented in a systematic manner. This model is grounded in the OBP principles outlined in the Dynamic Model of Occupation-Based Practice by Psillas and Stav [21] and is integrated with the occupational therapy process as defined by the OTPF-4 [7]. The service delivery process begins with a *referral system. The evaluation phase* consists of interviews focused on participants’ occupational histories, experiences, values, needs, and relevant contextual factors. Occupational profiles allow therapists to tailor interventions to individuals’ perspectives and backgrounds. The model prioritizes interventions based on the valued occupations of participants. This evaluation phase represents authentic occupation [21] by focusing on participants’ real-life activities and supporting the development of individualized treatment programs.

The *intervention phase* is guided by the OTPF-4 [7], including planning, implementation, and review. This process illustrates how occupation serves as both a means and an end, using the client’s occupations as a unifying framework for goal-setting, intervention planning, and outcome evaluation. It also demonstrates therapeutic intent [21], as occupational therapists synthesize evaluation findings with professional knowledge to design interventions that address clients’ needs. The intervention process aligns with the intervention plan and reflects meaningful and purposeful values [21] through the development and selection of treatment activities. The intervention focuses on what clients want, need, or are expected to do [32] in their daily lives. Additionally, this phase represents engaged participation [21] that is supported by the therapist to motivate clients to engage in activities with commitment and to encourage ongoing practice. Such engagement facilitates the achievement of established goals and enhances therapeutic outcomes.

The *outcome phase* highlights both successes and limitations of the intervention, identifying areas requiring modification or improvement. This process is essential for supporting participants in performing occupations independently and effectively. The model emphasizes participation in occupation and quality of life; therefore, selecting assessment tools that align with intervention goals is essential. The final phase of the model is the *transition or continuation phase,* in which the occupational therapist facilitates discharge planning and follow-up after participants return to the community. When challenges arise that hinder occupational performance, reentry into the evaluation phase may be considered.

An OBP service delivery model grounded in therapist preparation, client-centered goal setting, and outcome-focused intervention has the potential to enhance occupational performance, goal attainment, and quality of life for stroke survivors receiving community-based rehabilitation. Levels of understanding related to OBP and relevant knowledge were assessed before and after training. Prior to the training, the occupational therapist scored 10 out of 20, which increased to 17 out of 20 upon completion of the training. The increase in the occupational therapist’s scores suggests that the educational training contributed to improved understanding of OBP and related knowledge. This improvement indicates that structured training focusing on OBP concepts, assessment tools, and a well-designed service delivery framework may enhance the therapist’s conceptual clarity and readiness to apply OBP in her practice with clients in a community-based stroke rehabilitation setting.

### Evaluating the Implementation of an OBP Service Delivery Model

Seven chronic stroke survivors, with an average duration of 21.29 (SD 20.84) months post stroke, met the inclusion criteria for this study and were enrolled. Most participants were male, and their average age was 60.29 (SD 8.92) years, as presented in Table 1.

**Table 1.** Baseline demographic characteristics of clients (N=7).

Characteristics	Value
Sex, n (%)	
Male	5 (71)
Female	2 (29)
Dominant side, n (%)	
Right hand	7 (100)
Left hand	0 (0)
Type of stroke, n (%)	
Ischemic	4 (57)
Hemorrhage	3 (43)
Affected side, n (%)	
Right hemiparesis	4 (57)
Left hemiparesis	3 (43)
Others, mean (SD; minimum-maximum)	
Chronicity (mo)	21.29 (20.84; 6-58)
Age (y)	60.29 (8.92; 43-70)
MSET-10^a^ (score)	24 (5.35; 17-31)
BBS^b^ (score)	45.29 (9.05; 31-56)
9Q^c^ (score)	3 (2.16; 0‐6)

a MSET-10: The Mental State Examination Thai 10.

b BBS: Berg Balance Scale.

c 9Q: 9-Question Depression Assessment.

**Table 2.** Comparison of outcome measures within the group before and after the intervention.

Variable	Pretest mean (SD)	Posttest mean (SD)	Wilcoxon			Sign test
			*Z*	*P* value	Effect size (*r*)	*P* value
COPM^a^						
Performance	5.64 (1.89)	8.02 (1.79)	−2.38	.02^b^	0.64	.02^c^
Satisfaction	5.41 (1.96)	8.10 (1.63)	−2.38	.02^b^	0.64	.02^c^
GAS^d^	25.83 (3.08)	62.63 (6.11)	−2.37	.02^b^	0.63	.02^c^
WHOQoL-BREF^e^	83.14 (20.19)	110.14 (8.51)	−2.37	.02^b^	0.63	.02^c^

a COPM: Canadian Occupational Performance Measure.

b Significant at *P<.*05*, *Wilcoxon signed rank test was applied.

c Significant at *P<.*05*, *signed test was applied.

d GAS: goal attainment scaling.

e WHOQoL-BREF: World Health Organization Quality of Life–Brief Version.

As shown in Table 2, Wilcoxon signed-rank tests revealed significant improvements in COPM performance and satisfaction scores (*P*=.02; *r*=0.64), as well as GAS and WHOQoL-BREF scores (*P*=.02*; r*=0.63) with large effect sizes. Sign tests further demonstrated a consistent positive direction of change across participants for all outcome measures (*P*=.02). Comparisons of each outcome measure before and after the intervention for each participant, along with the sign test results, are presented in Tables S1 and S2 in Multimedia Appendix 2.

### Qualitative Reflections on Implementing an OBP Service Delivery Model

Furthermore, qualitative data obtained from an interview with the occupational therapist revealed two themes: (1) facilitating client-centered outcomes through OBP implementation and (2) enhancing professional confidence and supporting practical implementation of OBP in community-based practice.

The first theme, “facilitating client-centered outcomes through OBP implementation,” reflected the occupational therapist’s experiences using the OBP service delivery model to identify clients’ meaningful occupational needs and outcomes that were relevant to their everyday lives. The therapist expressed satisfaction with the results and described observable changes in clients’ daily occupations:

*I feel very happy with the results. Clients who previously attended our rehabilitation centers told me that they no longer needed to continue occupational therapy after years of rehabilitation. One client said, ‘I no longer need to come because I can now cook meals on my own and stay at home independently. My wife doesn’t need to leave work during the day to prepare my lunch. She can go to work in the morning knowing that I can manage on my own throughout the day.*

The therapist also described how focusing on clients’ meaningful occupations helped guide the intervention toward goals that were relevant to their everyday lives and living contexts:

*All of my clients told me what they wanted to do and achieve, which really helped me hear their voices. It really made sense to me that we need to focus on the clients’ needs, listen to them carefully, and help them find ways to engage in meaningful occupations. Meaningful occupations are what they truly want to do. I used to think it would be very difficult to do this in my setting, but it proved me wrong. Of course, we spent time fixing their muscle strength or other components. But now, I know that even a simple thing they want to do can be meaningful to them. For me, right now, just follow the clients’ occupational needs and support them.*

Furthermore, the second theme, “enhancing professional confidence and supporting practical implementation of OBP in community-based practice,” reflected the therapist’s experience of gaining confidence following learning about OBP and its application in clinical practice. The occupational therapist emphasized the value of training and the practical use of occupation-focused assessments and goal-setting processes:

*I really appreciate the service delivery process and the education training I received before implementing OBP with my clients. It was exactly what I had been looking for. The COPM and GAS, and goal-setting process are very practical, they help me understand what else I can do based on all information gathered from these assessments and individualized goals. I can clearly hear and see what my clients want. It is a great relief that, after several years of occupational therapy practice, I have found ways to help them achieve their occupational needs within their living contexts.*

The therapist further reflected on the practicality and feasibility of applying the OBP service delivery model within long-term, community-based occupational therapy services:

*It is going to be great if occupational therapists working in community-based rehabilitation services could use this service delivery model in their practice. If possible, I would encourage and support them in applying this model. Although it would take time to implement with clients, I would be happy to use it, even though it may not be applicable to every case. Still, I think it is practical and worthwhile.*

Overall, the findings suggested that the OBP service delivery model facilitated client-centered practice by supporting the occupational therapist in identifying and addressing clients’ meaningful occupational needs and aligning interventions with their real-life contexts and goals. The therapist perceived the model as practical and feasible for community-based rehabilitation services, although its implementation may require additional time and support and may not be applicable to every case. The occupational therapist further highlighted its potential for broader application in long-term community-based occupational therapy services.

## Discussion

### Principal Findings

This study contributes to the growing body of evidence supporting OBP for community-based stroke rehabilitation. In addition, it provides preliminary evidence for the feasibility of the proposed OBP service delivery model in rural community settings. Significant improvements were observed across all 4 outcome measures following implementation of the OBP service delivery model. The sign test showed that all participants improved in the same direction. Moreover, qualitative interviews with the occupational therapist who delivered the model provided additional insights into the feasibility of implementing the model. Therapist preparation through structured online OBP training and ongoing consultation with the research team were integral to supporting the consistent application of occupation-based principles throughout the intervention. Collaborative reflection between the therapist and the research team supported clinical reasoning, task grading, and adaptation of the intervention in response to participants’ evolving needs. The therapist also reported increased confidence and professional clarity following the implementation of OBP, suggesting that the model was perceived as practical and feasible for use in the rural community setting.

### Comparison to Prior Work

The occupational therapist reported that the occupation-centered service delivery process enhanced the ability to understand clients’ priorities and to translate assessment findings into meaningful, individualized goals. The therapist also perceived that OBP facilitated tangible gains for clients, leading to improved participation and quality of life. Consistent with Hovick and Provident [33], these findings stress that structured educational training can enhance occupational therapists’ professional confidence. Training that supports therapists in engaging in client-centered inquiry, formulating clinical questions, identifying and critically appraising relevant evidence, and integrating evidence into practice is highly valued and contributes to increased confidence in delivering interventions. Moreover, Krpalek et al [34] found that the delivery of occupational therapy services in the community is beneficial. Occupational therapists in the community play a crucial role focused on providing occupation-based interventions that facilitate client participation in their desired occupations.

The OBP service delivery model emphasized authentic engagement in daily life activities as both the means and the end of the intervention. By prioritizing occupations identified by participants through the COPM, the intervention aligned the occupation-based goals with stroke participants’ needs, values, and sociocultural contexts, including family support. Of 14 goals, the majority concentrated on self-care (n=8, 57%), followed by productivity (n=5, 36%) and leisure (n=1, 7%). Consistent with Kaunnil et al [19], Thai occupational therapists in physical rehabilitation settings most frequently addressed ADL, work, and instrumental ADL. This pattern indicates that ADL performance remains a central client priority and a core focus of occupational therapy intervention. Hence, these findings suggest that occupational therapists need to consider both participants’ needs and their environments when designing suitable goals and implementing OBP in rural settings.

According to Tiedemann et al [35], the lived experiences of patients with traumatic brain injury participating in an occupation-based community program reveal that engaging in meaningful and purposeful activities helps them form a new identity following their illness. This engagement enhances their self-image and provides them with a sense of accomplishment and belonging. The findings demonstrate the importance of implementing motivating activities to enhance patient engagement [35]. The design of occupation-based interventions may use various models as guides for developing occupation-based goals and treatment strategies that may differ from those used in this study.

In addition, the concurrent use of GAS enabled individualized goal setting and provided a sensitive method for capturing meaningful change across diverse occupational goals. GAS further emphasizes the effectiveness of integrating structured goal-setting methods into OBP. According to Skubik-Peplaski et al [14], OBP interventions enhanced goal attainment among rehabilitation clients. The combination of COPM and GAS provides a robust framework for ensuring that interventions remain client-driven and outcome-focused. Furthermore, Jung et al [36] demonstrated the utility of GAS in subacute stroke rehabilitation, highlighting its capacity to capture individualized change and clinically meaningful outcomes. When used alongside occupation-based assessments, GAS strengthens OBP by enabling therapists to systematically monitor progress, tailor interventions, and optimize occupational performance and satisfaction.

Furthermore, improvements in quality of life suggest that the OBP service delivery model may have broader psychosocial benefits beyond task performance, supporting holistic recovery and well-being. An and Kim [37] emphasize the potential of the WHOQOL for assessing enhancements in quality of life preintervention and postintervention by examining the effect of ADL dual-task training on stroke patients. Similarly, González-Bernal et al [38] use the WHOQOL to investigate quality of life among community-dwelling individuals with disabilities and to evaluate those who participated in a community occupational therapy program focused on the development of meaningful activities. Hence, using the WHOQOL alongside the COPM and GAS emphasizes a more holistic perspective on the client.

In this study, the outcomes reinforce prior evidence that OBP enhances functional performance and satisfaction with daily occupations [9,13,14]. The use of client-centered goal-setting tools, such as the COPM, appears to strengthen intervention effectiveness by aligning therapeutic strategies with clients’ personal aspirations [39]. While a recent review [16] cautions that more controlled trials are needed to confirm OBP’s superiority, the positive outcomes in this study highlight the potential of an OBP model tailored for long-term community rehabilitation. This was consistent with Jo and Kim [40], who used the Model of Human Occupation–based home modifications to encourage people with disabilities to participate in occupations that improve quality of life. This study revealed an increase in the time spent on ADL among community patients with physical disabilities. In contrast, Wasmuth et al [41] reported that, among community mental health populations, occupation-based interventions did not yield significant improvements in clinical recovery for individuals with severe mental illness; however, they were associated with positive changes in occupational participation. Hence, occupation-based approaches in community settings may be informed by different theoretical models while sharing a core emphasis on occupation as the central focus of intervention and on evaluating outcomes through occupational participation.

Qualitative feedback from occupational therapists emphasized the importance of client-centered goal setting in enabling stroke participants to achieve personally meaningful outcomes through OBP within their contexts. This finding is consistent with a previous study by Lee et al [42], which highlighted occupational therapists’ perceptions of the practical feasibility of OBP and the influence of contextual and client-related factors on its implementation. In addition, the findings reflected that the occupational therapists experienced increased confidence and professional satisfaction when delivering occupation-based intervention in real-life contexts. Similarly, Bolt et al [43] reported that, beyond promoting engagement in meaningful activities, OBP may contribute to occupational therapists’ professional satisfaction. Delivering intervention within real-life contexts may also enable occupational therapists to provide context-specific guidance to effectively address challenges in occupational performance [44]. Overall, the qualitative findings indicated positive perceptions of OBP, with the occupational therapists highlighting its contribution to client-centered practice, professional confidence, and practical implementation within the community rehabilitation context.

Recent evidence from a scoping review [45] indicates that OBP not only contributes to improvements in skill and functional performance but also enhances personal agency, intrinsic motivation, identity reconstruction, and social participation among stroke survivors. Through engagement in occupations that are personally meaningful and embedded within real-life contexts, occupational therapists facilitate intrinsic motivation, support problem-solving, and promote the re-establishment of a sense of self following stroke. This finding closely aligns with the present study, in which the OBP service delivery model prioritized individualized goal setting, contextual relevance, and active participation in everyday occupations within a rural community setting. Delivering OBP service delivery models in clients’ natural environments enabled stroke participants to reconnect with valued roles and routines, thereby fostering autonomy and sustained engagement in daily life. Consistent with the concept of evoking the powers of occupation [45], the positive outcomes observed in this study suggest that OBP is a key mechanism for translating therapeutic activities into meaningful occupational participation, particularly within a rural rehabilitation context in the Doi Lor community.

### Practical Implications

The findings of this study suggest that the OBP service delivery model may offer a practical and contextually responsive approach to community-based stroke rehabilitation, particularly in rural settings. The findings have several implications for occupational therapy practice and community rehabilitation services. First, the integration of therapist training, collaborative goal setting, meaningful occupation-based intervention, and systematic outcome evaluation may facilitate the implementation of OBP principles in occupational therapy practice. Second, the model may be particularly relevant to rural and resource-limited contexts where interventions need to be aligned with clients’ everyday contexts and available resources. The therapist’s feedback also suggests that the model may be adapted within existing community rehabilitation systems to support client-centered and occupation-based service delivery. Furthermore, the model provides a structured approach that may support long-term rehabilitation and participation among stroke survivors living in the community.

### Limitations

Several limitations should be acknowledged. First, the small sample size and lack of diversity among participants limit the generalizability of findings and reduce statistical power. Larger and more heterogeneous samples are recommended for future studies to strengthen external validity. Second, the absence of long-term follow-up restricts insights into the sustainability of intervention effects; longitudinal studies are needed to evaluate whether improvements in occupational performance and quality of life persist over time. Third, the study was conducted in community-based rehabilitation settings where access to other health care services is limited. Participants may not have received additional rehabilitation support outside this study, which reduced control over confounding variables but also highlights the importance of community-based OBP where alternative services are scarce. Fourth, this study developed an intervention tailored for community-dwelling stroke patients. Consequently, there may be limits to using the intervention when applying it to various populations or contexts. Using it in similar circumstances may be feasible. However, its application in various contexts may necessitate further study. Finally, the qualitative component involved a single occupational therapist who provided contextual information to inform the development of the OBP service delivery model and subsequently implemented the model and participated in the interview. Although the occupational therapist was not a codeveloper of the model, her prior involvement as a key informant and subsequent role as the implementing therapist may have influenced her perceptions and interview responses, introducing potential confirmation and response bias. Future studies should include multiple occupational therapists to capture diverse implementation experiences and reduce potential response bias.

### Conclusion

This preliminary study developed an OBP service delivery model grounded in occupational therapist training, collaborative goal setting, and structured outcome evaluation to support the long-term rehabilitation needs of stroke clients in a rural community setting. The findings suggest that the model may facilitate client-centered practice by aligning interventions with clients’ meaningful occupational needs, goals, and real-life contexts. The OBP service delivery model may offer a conceptual and practical framework for occupational therapists in long-term community care, with the potential to strengthen routine practice and promote holistic recovery for stroke clients in rural contexts. As such, the model may serve as a foundation for future development, implementation, and evaluation of OBP within community care systems.

## Supplementary material

10.2196/91118 Multimedia Appendix 1 Interview questions for the occupational therapist.

10.2196/91118 Multimedia Appendix 2 Quantitative outcome comparisons and qualitative content analysis.

## Abbreviations

ADL: activities of daily living
COPM: Canadian Occupational Performance Measure
GAS: goal attainment scaling
OBP: occupation-based practice
OTPF-4: Occupational Therapy Practice Framework: Domain and Process, Fourth Edition
WHOQOL: World Health Organization Quality of Life
WHOQOL-BREF: World Health Organization Quality of Life Scale–Brief Version
WHOQOL-BREF-THAI: Thai version of the World Health Organization Quality of Life–Brief Version
