# Supplementary material for: Developing and Implementing an Occupation-Based Practice Service Delivery Model for Stroke Survivors in a Rural Thai Community Rehabilitation Center: Mixed Methods Feasibility Study
Source: JMIR Form Res. 2026 Sep 21;10:e91118. doi: 10.2196/91118 (PMC13592663; doi:10.2196/91118)
Supplement: Supplementary file 1 [file formative-v10-e91118-s001.docx]

**Multimedia Appendix 1**

**Interview Questions**

1. Can you describe your overall experience of implementing the occupation-based practice (OBP) service delivery model in your workplace?
2. Since implementing OBP, what changes have you observed in your clients' occupational performance or daily lives? [Probe question: Can you provide specific examples? Can you describe a client whose progress particularly stands out?]
3. Has implementing OBP changed the way you make clinical decisions? If so, how?
4. Can you describe how education and training influenced your practice after implementing the occupation-based practice (OBP) service delivery model?
5. What aspects of the OBP service delivery model were most helpful during implementation?
6. What challenges did you encounter when implementing OBP, and how did you address them?
7. Looking back on your experience, what do you think has been the greatest benefit of implementing OBP?
