# Supplementary material for: Developing and Implementing an Occupation-Based Practice Service Delivery Model for Stroke Survivors in a Rural Thai Community Rehabilitation Center: Mixed Methods Feasibility Study
Source: JMIR Form Res. 2026 Sep 21;10:e91118. doi: 10.2196/91118 (PMC13592663; doi:10.2196/91118)
Supplement: Supplementary file 2 [file formative-v10-e91118-s002.docx]

**Multimedia Appendix 2**

**Table 1.** Comparison of the COPM scores and sign test between before and after the intervention for each participant

| Variable | COPM | | | | | |
| --- | --- | --- | --- | --- | --- | --- |
|  | performance  (Average) | | Sign test  (+/-/0) | satisfaction  (Average) | | Sign test  (+/-/0) |
|  | before | after |  | before | after |  |
| P1 | 2 | 5.33 | + | 1.67 | 5.67 | + |
| P2 | 5 | 7 | + | 4 | 7 | + |
| P3 | 5.5 | 7 | + | 5.5 | 7.5 | + |
| P4 | 6 | 9.33 | + | 6.67 | 9 | + |
| P5 | 6 | 7.5 | + | 6 | 7.5 | + |
| P6 | 8 | 10 | + | 7 | 10 | + |
| P7 | 7 | 10 | + | 7 | 10 | + |
| Median (IQR) | 6  (5-7) | 7.5  (7-10) | + | 6  (4-7) | 7.5  (7-10) | + |
| Mean  (SD) | 5.64  (1.89) | 8.02 (1.79) | + | 5.41  (1.96) | 8.10 (1.63) | + |

**Table 2.** Comparison of GAS and WHOQoL-BREF scores and sign test between before and after the intervention for each participant

| Variable | GAS  (T-score) | | Sign test  (+/-/tier) | WHOQoL-BREF  (Overall score) | | Sign test  (+/-/tier) |
| --- | --- | --- | --- | --- | --- | --- |
|  | before | after |  | before | after |  |
| P1 | 22.61 | 59.13 | + | 53 | 108 | + |
| P2 | 30 | 60 | + | 62 | 101 | + |
| P3 | 25.19 | 56.2 | + | 96 | 107 | + |
| P4 | 22.61 | 68.26 | + | 76 | 119 | + |
| P5 | 25.19 | 56.2 | + | 98 | 102 | + |
| P6 | 25.19 | 68.61 | + | 108 | 124 | + |
| P7 | 30 | 70 | + | 89 | 110 | + |
| Median  (IQR) | 25.19  (22.61-30) | 60  (56.2-68.61) | + | 89  (62-98) | 108  (102-119) | + |
| Mean  (SD) | 25.83 (3.08) | 62.63  (6.11) | + | 83.14 (20.19) | 110.14 (8.51) | + |

**Table 3.** Example of content analysis in this study.

| Domain | Key finding |
| --- | --- |
| Theme | Enhancing professional confidence and the practicality of through OBP implementation in community-based practice. |
| Categories | Value of education and implementation support |
| Codes | Appreciation of OBP training and service delivery process |
| Condensed meaning unit | Appreciated the OBP service delivery and training before implementation. |
| Unit of analysis of  an entire interview | “I really appreciate the service delivery process and the education training I received before implementing OBP with my clients. It was exactly what I had been looking for. The COPM and GAS, and goal-setting process are very practical, they help me understand what else I can do based on all information gathered from these assessments and individualized goals. I can clearly hear and see what my clients want. It is a great relief that, after several years of occupational therapy practice, I have found ways to help them achieve their occupational needs within their living contexts.” |
